# Does skin surface temperature variation account for Buruli ulcer lesion distribution?

**DOI:** 10.1371/journal.pntd.0007732

**Published:** 2020-04-20

**Authors:** Nicola K. Sexton-Oates, Andrew J. Stewardson, Arvind Yerramilli, Paul D. R. Johnson

**Affiliations:** 1 Department of Medicine, the University of Melbourne, Melbourne, Victoria, Australia; 2 Department of Infectious Diseases, Austin Health, Melbourne, Victoria, Australia; 3 Department of Infectious Diseases, Alfred Hospital and Central Clinical School, Monash University, Melbourne, Victoria, Australia; 4 Department of General Medicine, the Royal Melbourne Hospital, Melbourne, Victoria, Australia; Faculty of Science, Ain Shams University (ASU), EGYPT

## Abstract

**Background:**

Buruli ulcer is a necrotising infection of skin and soft tissue caused by *Mycobacterium ulcerans* (*M*. *ulcerans*). Buruli ulcer most often occurs on limbs, and it is hypothesized this is explained by direct exposure to the environment. However, even on exposed areas Buruli ulcer is not randomly distributed. *M*. *ulcerans* prefers an in vitro temperature of 30–33°C and growth is inhibited at higher temperatures. This study investigated whether variations in skin surface temperature distribution in healthy volunteers could partly account for Buruli ulcer lesion distribution.

**Methodology/Principal findings:**

In this observational study, a thermal camera (FLIR E8) was used to measure skin surface temperature at the sternal notch and at 44 predetermined locations on the limbs of 18 human participants. Body locations of high, middle and low Buruli ulcer incidence were identified from existing density maps of lesion distribution. Skin temperature of the three incidence location groups were compared, and differences in age and sex groups were also analysed.

We found an inverse relationship between skin temperature and lesion distribution, where high incidence locations were significantly cooler and low incidence locations significantly warmer (Kruskal-Wallis test p<0.0001). Linear mixed effects regression analysis estimated that skin surface temperature accounts for 22.0% of the variance in Buruli ulcer lesion distribution (marginal R-squared = 0.219) in the anterior location group, and 0.6% in the posterior group (marginal R-squared 0.006). Men had warmer upper and lower limbs than females (Mann-Whitney U test p = 0.0003 and p<0.0001 respectively).

**Conclusions/Significance:**

We have found an inverse relationship between skin temperature and Buruli ulcer lesion distribution, however this association is weak. Additional unknown factors are likely to be involved that explain the majority of the variation in Buruli lesion distribution.

## Author summary

Buruli ulcer is a destructive soft tissue infection caused by the bacterium *Mycobacterium ulcerans*. The precise mode of transmission remains unknown. One theory proposes that transmission occurs by direct contact with a contaminated environment. Lesions occur mostly on limbs, and it is hypothesized this is explained by direct exposure to the environment. However even on exposed areas, lesions are not randomly distributed. This study investigated whether skin surface temperature can partly explain Buruli ulcer lesion distribution. We measured the skin surface temperature of 18 healthy participants using a thermal camera and compared temperature distribution to the distribution of Buruli ulcer lesions investigated in a previously published study. We found that there is a negative correlation between skin temperature and Buruli ulcer lesion incidence. However, the association is weak and other factors e.g. clothing choice and insect biting patterns may explain the majority of Buruli ulcer lesion distribution.

## Introduction

Buruli ulcer is a necrotising cutaneous infection caused by the bacterium *Mycobacterium ulcerans* [1, 2]. Cases of Buruli ulcer have been reported in 33 countries, most of which are located in West and Central Africa [3], with children in these areas experiencing the majority of the disease burden [1]. Currently, there is a major outbreak occurring in south-eastern Australia on the Bellarine and Mornington peninsulas [4, 5]. Severe and/or untreated Buruli ulcer may result in contractures, deformity, permanent scarring, amputations and disabilities [6]. These can lead to social, educational and financial difficulties for those affected and their families, particularly in developing countries with limited access to modern therapy [7]. Seventy years on since the identification of *M*. *ulcerans* as the causative organism of Buruli ulcer, its transmission remains controversial. The disease only occurs in specific endemic locations but how exactly the infection is acquired in these regions is undetermined [5, 6].

There are several competing hypotheses concerning the transmission of *M*. *ulcerans*. Firstly, transmission may occur through direct contact with an environment contaminated with *M*. *ulcerans*, likely aided by minor cuts and abrasions sustained while working or playing outdoors [6]. Secondly, in south-eastern Australia there is increasing evidence that insects, particularly mosquitoes, may act as mechanical vectors to transmit the bacteria to humans [8]. Thirdly, *M*. *ulcerans* may be aerosolised from contaminated natural bodies of water, spread into the environment, then be inhaled and disseminated in the body [9]. The bacteria could then reactivate at cooler body sites [9, 10] as *M*. *ulcerans* prefers to grow in vitro at 30–33°C and growth is inhibited at higher temperatures [6], in a way analogous to *Mycobacterium leprae*, the causative organism of leprosy [11]. Human-to-human transmission is not thought to be of public health significance [12].

Buruli ulcer lesions are most common on limbs [1, 8, 10, 13–16]. We postulate that skin on these areas of the body is more likely to be exposed to a contaminated environment than other areas of the body, for example the trunk. Recently, computer-generated density maps of Buruli ulcer lesion distribution have been created by analysing the locations of 649 confirmed lesions in Victoria, Australia from 1998–2015 [10]. A highly non-random distribution was found, favouring distal limbs, particularly ankles, calves and elbows. Palms of the hands and soles of the feet were rarely affected. These findings are in keeping with the mosquito vector and direct contamination hypotheses of transmission, as most lesions occurred on commonly exposed areas of the body (i.e. limbs). However, palms of the hand and soles of the feet are rarely sites of Buruli ulcer lesions. This suggests an additional factor or factors are involved in the localisation of lesions beyond just direct environmental contact, for example, the preference of *M*. *ulcerans* to grow at cooler body sites, trauma, or insect bites [10]. This study aimed to investigate whether skin surface temperature distribution can explain variation in Buruli ulcer incidence in different regions of the body and between different demographics (i.e. age and sex categories).

## Methods

### Study design

This was an observational study using thermal imaging to investigate skin surface temperature in volunteer participants and enable comparison to published Buruli ulcer lesion distribution data. Measurements were undertaken in a single visit per participant at the Austin Hospital between April and June 2018. Eighteen volunteer participants were included in this study, recruited in age group cohorts: ≤15 (n = 2), 16–64 (n = 12) and ≥65 years of age (n = 4). This was to allow for comparison to published density maps of Buruli ulcer lesion distribution also categorised in these age groups. We aimed to recruit approximately 20 patients across the three age groups, based partly on time and resource availability. At the time the project was designed we were not aware of existing data on which to base a formal power calculation. We successfully recruited and studied 18 patients. We recruited a convenience sample of hospital staff, medical students, and family and friends of initial participants. Eligibility criteria included the ability to stand for 30 minutes and to be afebrile (<38°C) on the day of measurement.

### Data collection

A thermal camera (FLIR E8) was obtained to measure skin surface temperature from a distance of 30cm at the sternal notch and at 44 predetermined locations on the limbs (see S1 and S2 Appendices). The thermal camera used in this study had spatial resolution identified as sufficient for the evaluation of human skin temperature [17], and the lead researcher attended a 4 hour training course provided by the manufacturer (FLIR Systems, 18/03/2018). We measured locations specifically on the limbs as these areas are commonly affected by Buruli ulcer and postulated to be commonly exposed to the environment. However within these exposed areas there is variation in lesion prevalence, and so by measuring relative temperature at different limb locations we investigated whether this variation may explain the known non-random distribution of Buruli lesions. A measurement at the sternal notch was included to enable comparison of limb measurements to the trunk and hence comparison of our findings to previous research examining limb and trunk skin temperatures. Measurements were recorded in clinic rooms at the Austin Hospital to minimise variation in room temperature and surrounding surfaces, as these can affect skin surface temperature and thermal camera measurements. Two temperatures were recorded for each location, measured approximately 15 minutes apart. Thermal images of upper and lower limbs were also recorded from 1.5 and 3 metres.

Participants rested in the clinic room for approximately 10 minutes prior to measurement to minimise the effect of prior physical activity on skin surface temperature distribution. Participants also completed a questionnaire regarding age, sex and a number of medical conditions/medications known to affect skin surface temperature (see S3 Appendix). Room temperature was recorded using Aqua Systems ‘Wooden Wall Thermometer’. Core body temperature was measured using a temporal artery thermometer (Exergen TAT-5000) to ensure participants were afebrile. Core body temperature, sternal notch and left cubital fossa temperature measurements of a control, the investigator, were recorded at each session to examine the consistency of skin surface temperature measurements over time and with varying room and core body temperatures.

### Categorisation of body locations of high, medium and low Buruli ulcer incidence

**Fig 1:**
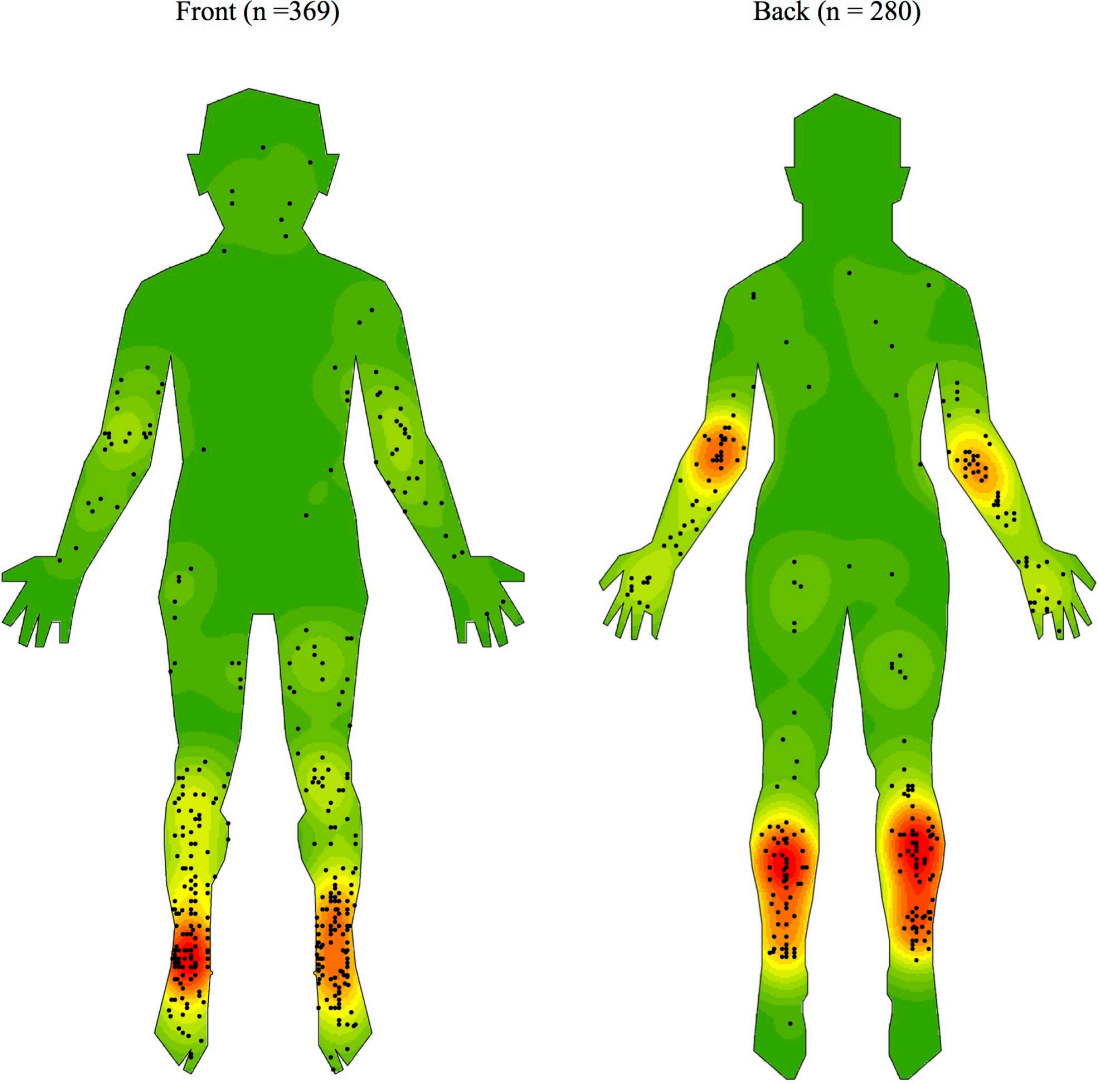
Density map of the distribution of Buruli ulcer lesions on front and back of human body templates generated using ArcGIS software version 10.3.1. [10].

Previously published density maps of Buruli ulcer lesion distribution (Fig 1) were created using a kernel density analysis and a 15-layer colour ramp from green (lowest density, 1/15) to red (highest density, 15/15) with an ‘equal interval’ classification system in ESRI ArcGIS, ArcMap (Economic and Social Research Institute, Redlands, USA, version 10.3.1) [10]. Anterior and posterior body maps were treated separately and thus separate kernel density analyses were performed for each. All medial and lateral lesions were allocated to the anterior body map in the study. The software’s default search radius (or bandwidth) was used which computes for each of the anterior and posterior input spatial datasets and aims to correct for any spatial outliers (A. Yerramilli, December 2019, personal communication). Using these published maps, density gradations were assigned to the body locations investigated in this study (Table 1). Given that the two maps (anterior ‘front’ and posterior ‘back’) were created using two separate kernel density analyses, further analyses in this study based upon assigned density gradations have treated anterior and posterior groups separately.

**Table 1:** Body locations with corresponding Buruli ulcer lesion density gradations, categorised into anterior and posterior location groups, and high, medium and low Buruli ulcer incidence groups.

Anterior Locations		Posterior Locations	
Location	Density gradation	Location	Density gradation
**High Buruli ulcer incidence**			
Anterior lower shin	8–15	Posterior upper calf	8–14
Medial malleolus	10–13	Posterior mid calf	14–15
Lateral malleolus	10–15	Posterior lower calf	7–12
		Elbow	10–12
**Medium Buruli ulcer incidence**			
Anterior mid shin	7–9	Posterior mid forearm	6–7
Anterior knee	6–7	Dorsum of hand	5–7
Dorsum of foot	7–9		
Anterior upper shin	5–7		
**Low Buruli ulcer incidence**			
Sternal notch	1	Posterior mid arm	3–4
Anterior mid thigh	1–3	Posterior mid thigh	2–3
Central palm	1–2	Popliteal fossa	3–5
Anterior mid forearm	2–3	Sole of foot	1
Cubital fossa	5		
Anterior mid arm	3–4		

Body locations of high Buruli ulcer incidence for this study have been defined as areas of lesion density in the highest third of density gradations, corresponding to gradations ≥ 11/15. Body locations of medium Buruli ulcer incidence have been defined as areas of lesion density in the middle third of density gradations, corresponding to gradations 6-10/15. Body locations of low Buruli ulcer incidence have been defined as areas with density gradation 1-5/15. If a location had a range of density gradations, the highest gradation was used to determine Buruli ulcer incidence category.

### Statistical analysis

The mean skin surface temperature of each body location was compared to the mean of all other locations combined in the corresponding anterior/posterior group using Mann-Whitney *U* tests. The median temperatures of the three Buruli ulcer incidence groups were compared using a Kruskal-Wallis test, with anterior and posterior group analyses performed separately. Differences in age and sex groups were also analysed using Kruskal-Wallis and Mann-Whitney *U* tests.

Additionally, we built a mixed-effects linear regression model for both anterior and posterior groups to quantify the association between temperature and incidence of Buruli ulcer in each body location. We accounted for the repeated temperature measurements by including random effects for participant (random intercept and slope) and for the potential impact of age by including a random slope for age.

The regression model was built using R, version 3.4.4 (R Foundation for Statistical Computing, Vienna, Austria). All other analyses were performed using GraphPad Prism version 7.04.

### Ethical statement

This study was approved by the Austin Health Human Research Ethics Committee. Reference number: HREC/17/Austin/578. Written consent was obtained from each participant.

## Results

### Participant cohort analysis

Eighteen participants were included. The mean age was 38.7 years (range = 11.8–77.3, IQR = 24.7–64.4). Nine participants were male (50%) and 9 female (50%). In the ≤ 15 years age group (n = 2), there were 2 (100%) female participants. In the 16–64 years age group (n = 12), there were 6 (50%) females and 6 (50%) males. In the ≥ 65 years age group (n = 4), there was 1 (25%) female and 3 (75%) males.

Of the 18 participants, 4 (22%) reported having experienced chilblains, 1 (6%) peripheral vascular disease, 1 (6%) suspected Raynaud’s phenomenon, 1 (6%) low-functioning thyroid on thyroxine with normal TSH (thyroid-stimulating hormone) levels, and 1 (6%) taking a blood pressure medication (Irbesartan). No participants reported having diabetes for > 5 years, a high-functioning thyroid, neuropathy, sunburn or taking migraine medication.

### Grouping of left and right measurements

With the exception of the sternal notch, measurements were recorded on both sides of the body for each location, e.g. left anterior knee and right anterior knee. As there was no significant difference between left and right measurements by Mann-Whitney *U* test (p = 0.4844), these groups were combined to give 23 body locations for reporting of mean skin surface temperature and further analysis.

### Mean skin surface temperature in high, medium and low Buruli ulcer incidence locations

**Fig 2:**
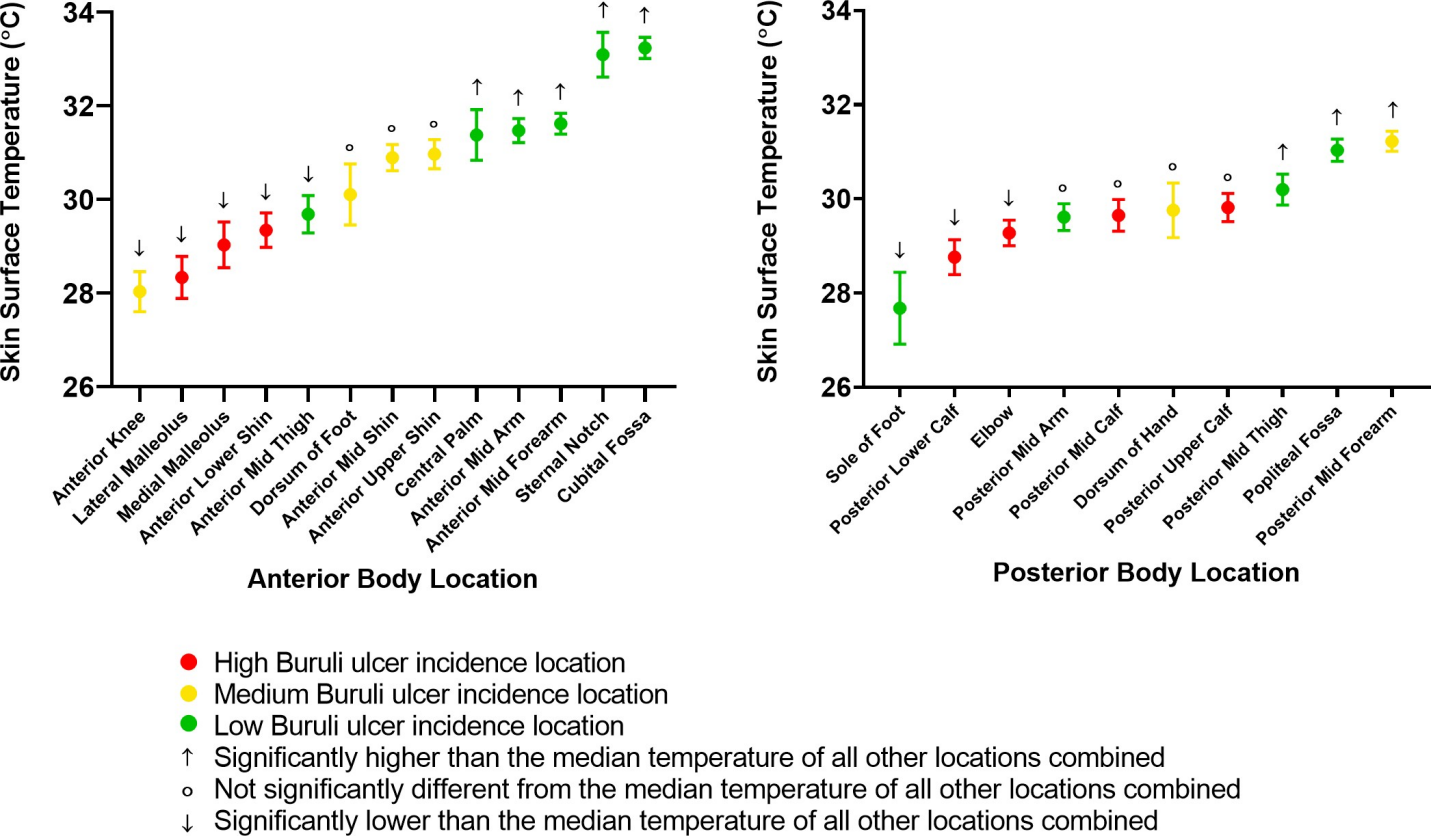
Mean skin surface temperature with 95% confidence interval for each body location, shown in separate anterior location (left) and posterior location (right) facets. Results of comparison to median skin surface temperature of all other locations combined (in the corresponding anterior/posterior group) by Mann-Whitney *U* test are also shown (p values from left to right: p<0.0001, p<0.0001, p<0.0001, p<0.0001, p = 0.0006, p = 0.4891, p = 0.1363, p = 0.0833, p<0.0001, p<0.0001, p<0.0001, p<0.0001, p<0.0001, p<0.0001, p<0.0001, p = 0.0004, p = 0.1349, p = 0.3569, p = 0.7009, p = 0.7731, p = 0.042, p<0.0001, p<0.0001).

**Fig 3:**
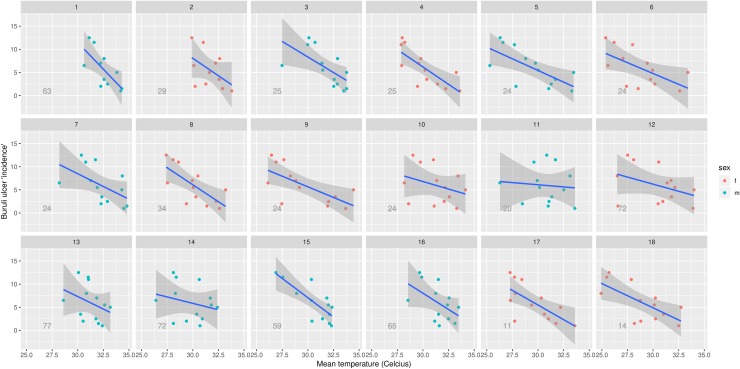
**Scatterplot of mean temperature and Buruli ulcer incidence gradation for each anterior body location, overlayed by a line of best fit.** Each facet represents data from a single participant. Each point represents a body location. The line of best fit was computed using linear regression, and the shaded region represents the 95% confidence interval for the line of best fit. The integer projected on each facet corresponds to the age (in years) of the participant.

**Fig 4:**
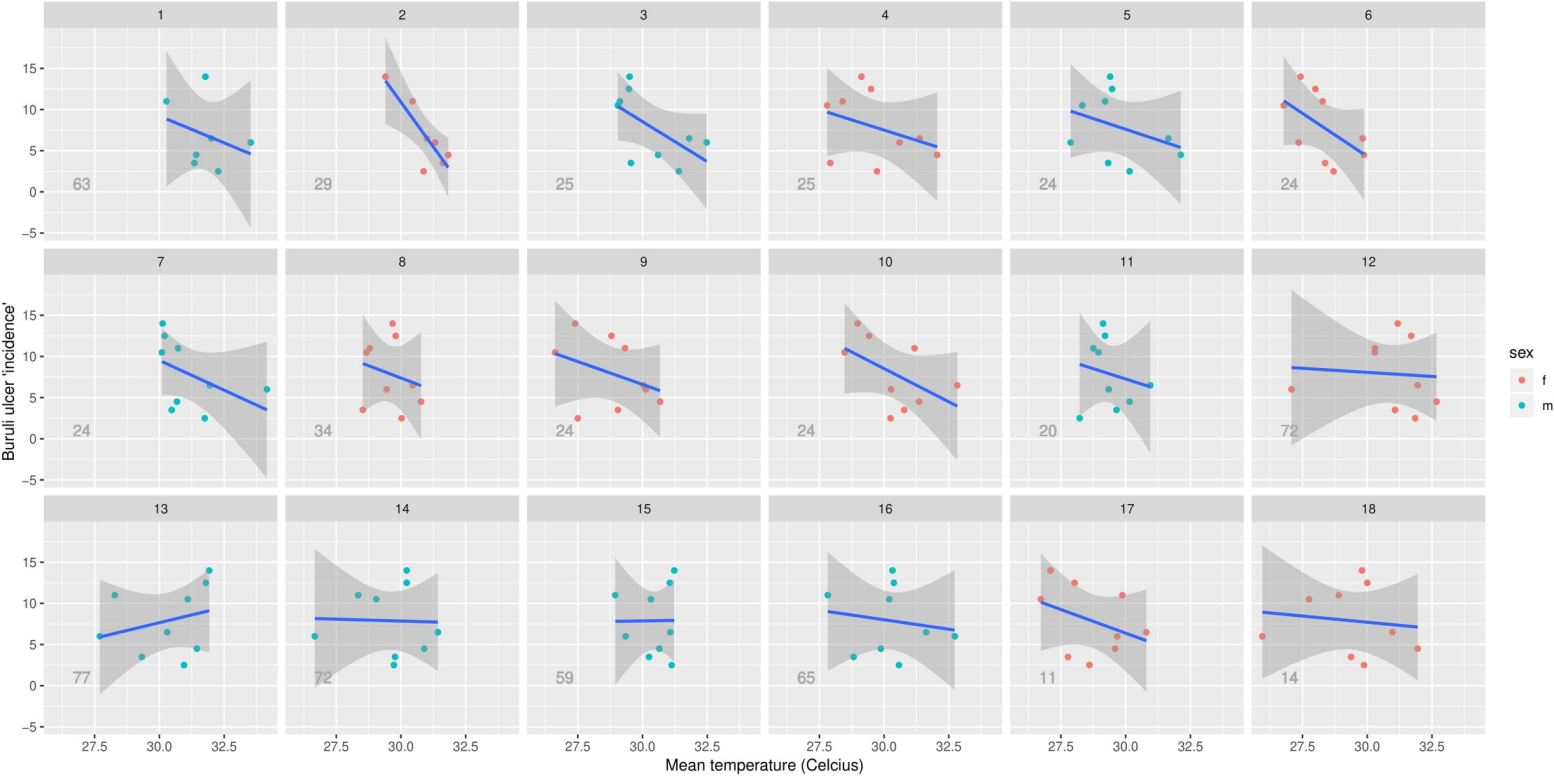
Scatterplot of mean temperature and Buruli ulcer incidence gradation for each posterior body location, overlayed by a line of best fit. Each facet represents data from a single participant. Each point represents a body location. The line of best fit was computed using linear regression, and the shaded region represents the 95% confidence interval for the line of best fit. The data points for the location ‘sole of foot’ have been excluded. The integer projected on each facet corresponds to the age (in years) of the participant.

Participant skin surface temperature measurements ranged from 22.6 to 35.3°C, with a mean of 30.1°C (Table 2). Skin surface temperature data was not normally distributed (D’Agostino-Pearson normality test p<0.0001). Cubital fossa was the location of highest mean skin surface temperature (33.2°C) and sole of foot the location of lowest mean skin surface temperature (27.7°C) (Table 2 and Fig 2). Overall, the three incidence groups were found to have significantly different median temperatures (Kruskal-Wallis test p<0.0001 for both anterior and posterior groups). In the anterior location group, the high incidence group was the coolest, the low incidence group the warmest, and the medium incidence group fell in between (median 28.7, 31.9 and 30.0°C respectively). This is reflected in Fig 2 (anterior locations), 3 (Fig 3) and 4 (Fig 4), which show a visually-apparent negative correlation between Buruli ulcer incidence and mean skin surface temperature. In the posterior location group, the high incidence group was also the coolest, however the medium incidence group was the warmest and the low incidence group fell in between (median 29.4, 30.9 and 30.2°C respectively). On the anterior aspect of the body, the negative correlation is supported by linear mixed effects regression analysis, which estimates that skin surface temperature accounts for 22.0% of the variance in Buruli ulcer lesion distribution (marginal R-squared = 0.219). Additionally, for each one-degree (Celsius) increase in the temperature of a body location there is a 0.79 (95% CI, 0.63–0.96) reduction in incidence category of that part of the anterior aspect of the body. On the posterior aspect of the body, skin surface temperature accounted for 0.6% of the variance in Buruli ulcer lesion distribution (marginal R-squared = 0.006). For each one-degree (Celsius) increase in the temperature of a body location there is a 0.17 (95% CI, -0.25–0.59) reduction in incidence category of that part of the posterior aspect of the body. As a sensitivity analysis, we repeated the posterior body surface analysis without the (outlier) sole of foot measurements. The same model then accounted for 5.2% of variance in Buruli ulcer distribution, with each one-degree (Celsius) increase in the temperature associated with a 0.57 (95% CI, 0.32–0.81) reduction in incidence category.

**Table 2:** Mean and range of temperature recordings.

Measurement	Mean (°C)	Range (°C)
**Anterior and Posterior Locations**	30.1	22.6–35.3
**Anterior Locations**		
Sternal notch	33.1	30.7–34.5
Central palm	31.4	25.4–35.2
Mid forearm	31.6	30.0–33.9
Cubital fossa	33.2	31.1–35.3
Mid arm	31.5	29.2–33.4
Dorsum of foot	30.1	23.8–35.0
Medial malleolus	29.0	24.7–32.2
Lateral malleolus	28.3	24.2–32.0
Lower shin	29.4	26.6–32.0
Mid shin	30.9	28.5–32.9
Upper shin	31.0	27.5–32.9
Knee	28.0	25.0–32.1
Mid thigh	29.7	26.7–32.8
**Posterior Locations**		
Dorsum of hand	29.8	25.7–34.7
Mid forearm	31.2	29.0–33.6
Elbow	29.3	27.0–32.0
Mid arm	29.6	27.4–32.2
Sole of foot	27.7	22.6–32.7
Lower calf	28.8	25.8–31.6
Mid calf	29.7	26.2–32.4
Upper calf	29.8	27.1–32.5
Popliteal fossa	31.0	28.1–32.9
Mid thigh	30.2	26.8–32.8
**Additional Measurements**		
Control sternal notch	33.9	32.5–35.3
Control left cubital fossa	33.7	32.5–34.7
Control core body temperature	36.6	36.3–36.9
Room temperature	21.1	20.0–25.0
Participant core body temperature	36.5	36.0–37.0

With respect to the 7 high Buruli ulcer incidence locations, 5 (71%) had median skin surface temperature significantly lower than the median of all other locations combined in the corresponding anterior/posterior group when testing with Mann-Whitney U tests (Fig 2). These locations were elbow, anterior lower shin, lateral malleolus, medial malleolus, and posterior lower calf. Two (29%) high Buruli ulcer incidence locations, posterior mid calf and posterior upper calf, did not have median temperature significantly different from the median of all other locations combined. No high Buruli ulcer incidence locations had mean temperatures significantly higher than the mean of all other locations combined. Notably, one low incidence location was also within the same temperature range as high lesion density locations; posterior mid arm. Locations over joints were found to have a significantly lower median temperature than non-joint locations (median 29.90 and 30.40°C respectively, p = 0.0008).

### Comparison by age group and sex

Analysis of skin surface temperatures by sex and age group are shown in Table 3. All male and all female measurements were compared using a Mann-Whitney *U* test. The male group had a significantly higher skin surface temperature overall than the female group (p<0.0001), with a mean of 30.6°C compared to 29.6°C respectively. When comparing all upper limb measurements, males had a significantly higher mean skin surface temperature than females (31.5 and 30.8°C respectively, p = 0.0003). When comparing all lower limb measurements, males also had a significantly higher mean skin surface temperature than females (30.2 and 28.9°C respectively, p<0.0001).

**Table 3:** Comparison of mean skin surface temperature by age group and sex.

Location	Mean skin temperature (°C)				p value
	**Male**		**Female**		
All locations	30.6		29.6		<0.0001 (Mann-Whitney U)
Upper limb	31.5		30.8		0.0003 (Mann-Whitney U)
Lower limb	30.2		28.9		<0.0001 (Mann-Whitney U)
	**≤15 years**	**16–64 years**		**≥65 years**	
All locations	28.1	30.3		30.3	<0.0001 (Kruskal-Wallis)
	**<65 years**		**≥65 years**		
Mid arm	30.6		30.4		0.433 (Mann-Whitney U)

The skin surface temperature measurements of the three age groups were compared using a Kruskal-Wallis test and found to be significantly different (p<0.0001). The ≤15 age group had an overall mean skin surface temperature of 28.1°C (range 23–33.6), the 16–64 age group 30.3°C (range 24–35.3) and the ≥ 65 age group 30.3°C (range 22.6–34.7). However, these data should be considered with caution as we were only able to recruit 2 participants for the ≤15 age group.

To enable comparison to previously published work, anterior and posterior mid arm temperatures combined were compared between those <65 years and those ≥65 years. No significant difference was found by Mann-Whitney *U* test (p = 0.433, means 30.59 and 30.37°C respectively).

### Control measurements

Measurements of the constant control’s core body temperature, sternal notch and left cubital fossa skin temperature varied throughout the data collection period (see Table 1). Sternal notch ranged from 32.5–35.3°C, left cubital fossa 32.5–34.7°C, and core body temperature 36.3–36.9°C. The constant control was taking the oral contraceptive pill during the measurement period and so menstrual cycle hormonal changes were not expected to affect core body temperature.

## Discussion

We have found that overall, the three Buruli ulcer incidence location groups derived from previously published work had significantly different median skin surface temperatures. In the anterior location group analysis, the highest incidence group had the coolest median temperature, the lowest incidence group had the highest median temperature, and the middle incidence group fell in between. In addition, the linear mixed effects regression analysis for the anterior group estimated that skin surface temperature accounts for 22.0% of the variance in Buruli ulcer lesion distribution (marginal R-squared = 0.219). This generally supports a previously stated hypothesis that Buruli ulcer lesions occur preferentially on areas of relatively lower skin temperature. However in the posterior group, skin surface temperature accounted for only 0.6% of the variance in Buruli ulcer lesion distribution (marginal R-squared = 0.006). Additionally, one low incidence area (posterior mid arm) was within the same skin surface temperature range as the high incidence group, and the coolest region of all, sole of foot, is rarely affected by Buruli ulcer.

Buruli ulcer lesion distribution has been found to differ between men and women, and between age groups. For example, men have been found to be more likely than females to have lesions on upper limbs, and less likely to have lesions on lower limbs [10, 15]. Those ≥65 years of age have been found to be less likely to have a lesion on the arm and shoulder than those <65 [10]. We investigated whether these differences between age and sex correlated with differences in skin surface temperature distribution. We found that men had significantly higher skin surface temperatures than females for both upper and lower limbs. There was no significant difference in mid arm measurements found between those <65 and those ≥65. As such, we conclude that differences in skin surface temperature distribution found in this study do not account for differences in Buruli ulcer lesion distribution between age and sex groups. We have also explored the relationship between joint locations and skin surface temperature, as Buruli ulcer is known to have a predilection for joints, for example as reported in a study by Boyd et al where 40% of lesions occurred over joints [15]. In our study, joint locations were found to have a significantly cooler median temperature than non-joint locations (median 29.90 and 30.40°C respectively, Mann-Whitney *U* test p = 0.0008).

Our findings add some support to the aerosol-dissemination and reactivation hypothesis of transmission as we found a negative correlation between skin surface temperature and Buruli ulcer lesion distribution. However, the association is not consistent between anterior and posterior groups, and there are important exceptions (e.g. sole of foot) where the relationship breaks down. With respect to the mosquito vector hypothesis, skin temperature may influence mosquito biting patterns and hence Buruli ulcer lesion distribution. A future direction of research may be the direct investigation of the biting patterns of mosquitoes, particularly of species hypothesised to mechanically transmit *M*. *ulcerans* in south-eastern Australia.

The relationship between skin surface temperature and Buruli ulcer lesion incidence has been investigated in a previous study by Dezemon et al, where a visual inverse correlation was found, in keeping with our findings [18]. Several studies have examined skin temperature distribution more generally and found that the trunk is warmer than limbs, and proximal areas of limbs warmer than distal areas [19, 20]. The results from this study are consistent with these observations, finding that the sternal notch on the trunk had a mean skin surface temperature higher than 21 of 22 measured limb locations. In addition, the mean foot measurements were colder than mean mid thigh measurements, and the mean dorsal hand temperature was colder than mean posterior mid arm. In contrast, the mean temperature for palm of hand was warmer than that of the anterior mid arm.

The location ‘sole of foot’ was an outlier in this study. A number of factors may have contributed to this, including the influence of floor temperature on sole of foot surface temperature. Participants were wearing open summer footwear (e.g. sandals) during data recording to minimise this influence, however during the taking off and on of shoes to measure temperature, feet may have briefly come into contact with the floor. Secondly, soles of feet may not be as commonly exposed to the environment and biting insects as other limb locations due to being covered by footwear. Thirdly, the palms of the hand and soles of the feet are covered by glabrous skin, which is hairless, more heavily keratinised and has a thicker epidermis than most other areas of the body surface [21]. These factors may make these regions less hospitable to *M*. *ulcerans* and/or a less likely place for mosquito bites to occur, resulting in fewer lesions in these areas, and contribute to rendering ‘sole of foot’ an outlier. ‘Sole of foot’ was categorised into the posterior location group, and its outlier status likely contributes to the weaker negative correlation found in the posterior group as compared to the anterior location group.

The validity of thermal camera measurements and control of the factors which may influence them are important to consider. Thermal cameras have been used to record skin surface temperature in both disease and non-disease states [22]. We have used a thermal camera identified as appropriate for clinical research in humans [17], and believe our method of measurement (on-the-spot readings from a distance of 30cm, as opposed to taking measurements from a thermal image at a greater distance using a software program) optimises the validity and consistency of temperature readings. This conclusion draws from the training course run by FLIR Systems. Surfaces within the room and properties of the measured surface (in this case, skin) affect thermal camera measurements [23]. We controlled these variables by adjusting the thermal camera emissivity setting to 0.98, appropriate for human skin [23], and conducting the measurements in clinic rooms at the Austin hospital containing similar surfaces.

Limitations of this study include the fact that specific points were used to represent temperature for a larger area, e.g. olecranon fossa for elbow. Within a defined area there are often multiple smaller areas of differing temperature (see S4 Appendix), and as such the selected measurement points may not accurately represent the temperature of the larger area. In addition, the number of measurement locations may limit the generalisability of our findings to the whole body. A further limitation is the variance in room temperature (20–25°C) as this may have affected skin surface temperature and has not been taken into account in our analyses. The variation of skin surface temperature of the control was 2.8°C for the sternal notch and 2.2°C for the cubital fossa, and may be due to genuine fluctuation in skin surface temperature or inconsistent measurement. An additional limitation is that this study included a small number of participants with conditions likely to affect skin surface temperature e.g. peripheral vascular disease [17]. Participants rested for at least 10 minutes prior to measurements to minimise the effect of prior physical activity, however other factors that may influence skin surface temperature and its distribution (e.g. emotional state) are difficult to ascertain and were not controlled for. Lastly, the non-Gaussian distribution of the data and correlation between adjacent regions of the body may limit the appropriateness of the linear regression model.

In conclusion, we have found that there is an inverse relationship between skin surface temperature in healthy volunteers and previously published Buruli ulcer lesion distribution. However relative skin temperature appears to be only weakly associated with Buruli lesion distribution, meaning that 78 to 99% of the clinically observed non-random distribution is likely to be explained by other factors such as clothing choice, skin trauma and targeting behaviour by insects.

## Supporting information

S1 Appendix Skin temperature measurement procedure. This image displays the thermal camera screen during measurement. The crosshairs (labelled Sp1) indicate the point of measurement, and the temperature reading in the top left hand corner of the image shows the measured temperature of that area.
(JPG)

S2 Appendix Skin temperature measurement locations. (PNG)

S3 Appendix Study questionnaire. (DOCX)

S4 Appendix Selection of participant thermographs. a) Anterior lower limbs, b) Anterior upper limbs, c) Posterior lower limbs, d) Posterior upper limbs.
(PNG)

S5 Appendix STROBE checklist. (DOC)

## References

[1] Johnson PDR, Stinear T, Small PLC, Pluschke G, Merritt RW, Portaels F, et al Buruli Ulcer (M. ulcerans Infection): New Insights, New Hope for Disease Control. PLoS Medicine. 2005;2(4):e108 10.1371/journal.pmed.0020108 15839744 PMC1087202

[2] MacCallum P, Tolhurst JC, Buckle G, Sissons HA. A new mycobacterial infection in man. J Pathol Bacteriol. 1948;60(1):93–122. 18876541

[3] World Health Organisation. Buruli ulcer (Mycobacterium ulcerans infection) Fact Sheet 2017 [updated 02/2017. Available from: http://www.who.int/mediacentre/factsheets/fs199/en/.

[4] Carson C, Lavender CJ, Handasyde KA, O'Brien CR, Hewitt N, Johnson PD, et al Potential wildlife sentinels for monitoring the endemic spread of human Buruli ulcer in South-East Australia. PLoS Negl Trop Dis. 2014;8(1):e2668 10.1371/journal.pntd.0002668 24498452 PMC3907424

[5] Loftus MJ, Tay EL, Globan M, Lavender CJ, Crouch SR, Johnson PDR, et al Epidemiology of Buruli Ulcer Infections, Victoria, Australia, 2011–2016. Emerg Infect Dis. 2018;24(11):1988–97. 10.3201/eid2411.171593 30334704 PMC6199991

[6] Merritt RW, Walker ED, Small PL, Wallace JR, Johnson PD, Benbow ME, et al Ecology and transmission of Buruli ulcer disease: a systematic review. PLoS Negl Trop Dis. 2010;4(12):e911 10.1371/journal.pntd.0000911 21179505 PMC3001905

[7] Asiedu K, Etuaful S. Socioeconomic implications of Buruli ulcer in Ghana: a three-year review. Am J Trop Med Hyg. 1998;59(6):1015–22. 10.4269/ajtmh.1998.59.1015 9886216

[8] Lavender CJ, Fyfe JA, Azuolas J, Brown K, Evans RN, Ray LR, et al Risk of Buruli ulcer and detection of Mycobacterium ulcerans in mosquitoes in southeastern Australia. PLoS Negl Trop Dis. 2011;5(9):e1305 10.1371/journal.pntd.0001305 21949891 PMC3176747

[9] Hayman J. Postulated epidemiology of Mycobacterium ulcerans infection. Int J Epidemiol. 1991;20(4):1093–8. 10.1093/ije/20.4.1093 1800409

[10] Yerramilli A, Tay EL, Stewardson AJ, Kelley PG, Bishop E, Jenkin GA, et al The location of Australian Buruli ulcer lesions-Implications for unravelling disease transmission. PLoS Negl Trop Dis. 2017;11(8):e0005800 10.1371/journal.pntd.0005800 28821017 PMC5584971

[11] Scollard DM, Adams LB, Gillis TP, Krahenbhul JL, Truman RW, Williams DL. The Continuing Challenges of Leprosy. Clinical Microbiology Reviews. 2006;19(2):338–81. 10.1128/CMR.19.2.338-381.2006 16614253 PMC1471987

[12] O'Brien DP, Wynne JW, Buultjens AH, Michalski WP, Stinear TP, Friedman ND, et al Exposure Risk for Infection and Lack of Human-to-Human Transmission of Mycobacterium ulcerans Disease, Australia. Emerg Infect Dis. 2017;23(5):837–40. 10.3201/eid2305.160809 28418294 PMC5403060

[13] Pouillot R, Matias G, Wondje CM, Portaels F, Valin N, Ngos F, et al Risk factors for Buruli ulcer: a case control study in Cameroon. PLoS Negl Trop Dis. 2007;1(3):e101 10.1371/journal.pntd.0000101 18160977 PMC2154388

[14] Bratschi MW, Bolz M, Minyem JC, Grize L, Wantong FG, Kerber S, et al Geographic distribution, age pattern and sites of lesions in a cohort of Buruli ulcer patients from the Mape Basin of Cameroon. PLoS Negl Trop Dis. 2013;7(6):e2252 10.1371/journal.pntd.0002252 23785529 PMC3681622

[15] Boyd SC, Athan E, Friedman ND, Hughes A, Walton A, Callan P, et al Epidemiology, clinical features and diagnosis of Mycobacterium ulcerans in an Australian population. Med J Aust. 2012;196(5):341–4. 10.5694/mja12.10087 22432674

[16] Hospers IC, Wiersma IC, Dijkstra PU, Stienstra Y, Etuaful S, Ampadu EO, et al Distribution of Buruli ulcer lesions over body surface area in a large case series in Ghana: uncovering clues for mode of transmission. Trans R Soc Trop Med Hyg. 2005;99(3):196–201. 10.1016/j.trstmh.2004.05.004 15653121

[17] Hildebrandt C, Raschner C, Ammer K. An overview of recent application of medical infrared thermography in sports medicine in Austria. Sensors (Basel). 2010;10(5):4700–15. 22399901 10.3390/s100504700 PMC3292141

[18] Zingue D, Bouam A, Tian RBD, Drancourt M. Buruli Ulcer, a Prototype for Ecosystem-Related Infection, Caused by Mycobacterium ulcerans. Clinical Microbiology Reviews. 2018;31(1):01. 10.1128/CMR.00045-17 PMC5740976 29237707

[19] Zhu WP, Xin XR. Study on the distribution pattern of skin temperature in normal Chinese and detection of the depth of early burn wound by infrared thermography. Ann N Y Acad Sci. 1999;888:300–13. 10.1111/j.1749-6632.1999.tb07964.x 10842641

[20] Kolosovas-Machuca ES, Gonzalez FJ. Distribution of skin temperature in Mexican children. Skin Res Technol. 2011;17(3):326–31. 10.1111/j.1600-0846.2011.00501.x 21338404

[21] Young B, O'Dowd G, Woodford P. Skin Wheater's Functional Histology 6th ed Philadelphia, USA: Churchill Livingstone, an imprint of Elsevier Ltd; 2014 p. 159–79.

[22] Jones BF. A reappraisal of the use of infrared thermal image analysis in medicine. IEEE Trans Med Imaging. 1998;17(6):1019–27. 10.1109/42.746635 10048859

[23] Systems F. User's Manual FLIR Ex Series. 2007.

