# Supplementary figures and images for: Does skin surface temperature variation account for Buruli ulcer lesion distribution?
Source: PLoS Negl Trop Dis. 2020 Apr 20;14(4):e0007732. doi: 10.1371/journal.pntd.0007732 (PMC7192506; doi:10.1371/journal.pntd.0007732)

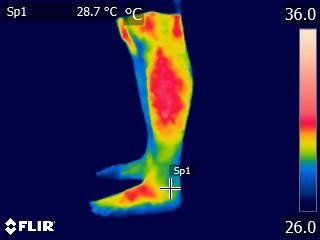

Supplement: S1 Appendix — This image displays the thermal camera screen during measurement. The crosshairs (labelled Sp1) indicate the point of measurement, and the temperature reading in the top left hand corner of the image shows the measured temperature of that area. (JPG) [file pntd.0007732.s001.jpg]

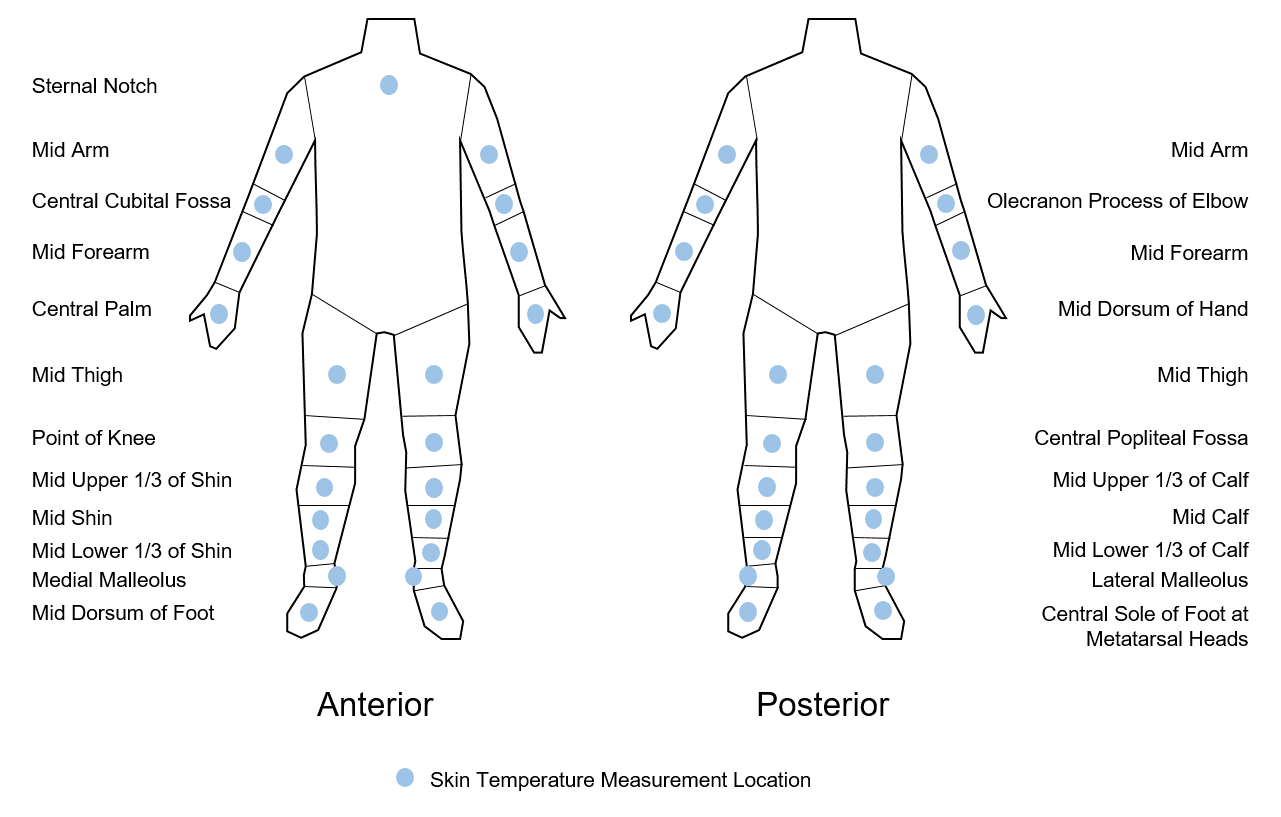

Supplement: S2 Appendix — (PNG) [file pntd.0007732.s002.png]

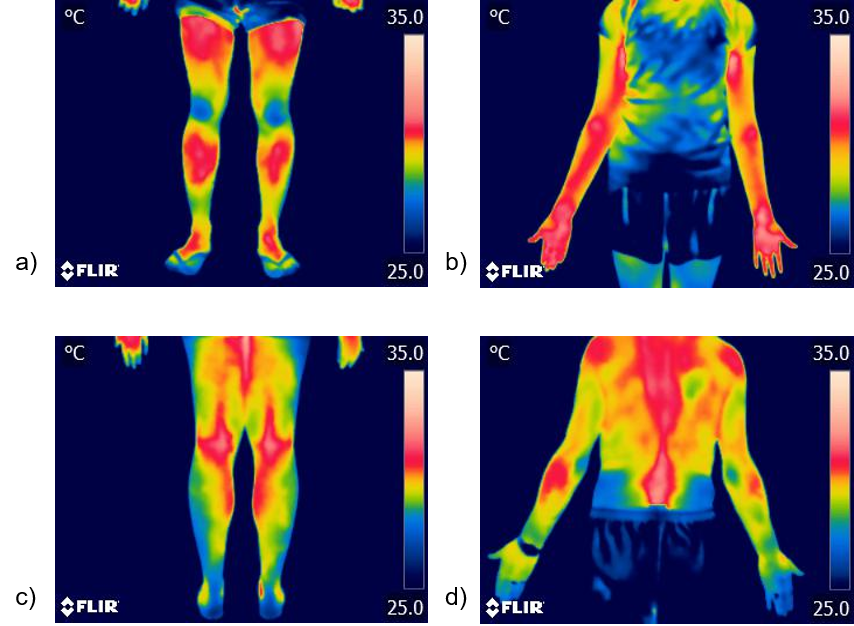

Supplement: S4 Appendix — a) Anterior lower limbs, b) Anterior upper limbs, c) Posterior lower limbs, d) Posterior upper limbs. (PNG) [file pntd.0007732.s004.png]
