# Supplementary material for: Does skin surface temperature variation account for Buruli ulcer lesion distribution?
Source: PLoS Negl Trop Dis. 2020 Apr 20;14(4):e0007732. doi: 10.1371/journal.pntd.0007732 (PMC7192506; doi:10.1371/journal.pntd.0007732)
Supplement: S3 Appendix — (DOCX) [file pntd.0007732.s003.docx]

SKIN THERMOGRAPHY QUESTIONNAIRE

Name:

Age (in years and months):

Sex: Female Male Other

1. **Have you been diagnosed with any of the following conditions?**

| **CONDITION** | **YES** | **NO** | **UNSURE** |
| --- | --- | --- | --- |
| Peripheral vascular disease |  |  |  |
| Diabetes (for more than 5 years) |  |  |  |
| A high-functioning thyroid |  |  |  |
| A low-functioning thyroid |  |  |  |
| Raynaud’s disease |  |  |  |
| Chilblains |  |  |  |
| Neuropathy |  |  |  |

1. **Do you currently take any of the following medications?**

| **MEDICATION** | **YES** | **NO** | **UNSURE** |
| --- | --- | --- | --- |
| Blood pressure medication (e.g. Beta blockers, Calcium channel inhibitors) |  |  |  |
| Migraine medication |  |  |  |

If so, please list the medications:

1. **Are you currently sunburnt?** Please tick one box.

YES NO UNSURE
